# Preoperative gemcitabine based chemo-radiotherapy in locally advanced non metastatic pancreatic adenocarcinoma

**DOI:** 10.1186/1755-7682-2-7

**Published:** 2009-03-27

**Authors:** Doaa W Maximous, Mostafa E Abdel-Wanis, Mohammed I El-Sayed, Alaa A Abd-Elsayed

**Affiliations:** 1 Department of surgical oncology, South Egypt Cancer Institute, Assiut University, Assiut, Egypt; 2 Department of radiotherapy, South Egypt Cancer Institute, Assiut University, Assiut, Egypt; 3 Public Health and Community Medicine Department, Faculty of Medicine, Assiut University, Egypt

## Abstract

**Introduction:**

Almost 30% of patients with pancreatic cancer have locally advanced tumours in absence of distant metastasis. Surgical resection is often contraindicated. The combination of gemcitabine with concurrent radiation therapy is a promising new approach that is being investigated for treating patients' unresectable pancreatic cancer. This work aims at assessing the efficacy of preoperative gemcitabine based chemo-radiotherapy in increasing the resectability rate for patients' locally advanced pancreatic cancer.

**Patients and methods:**

From March 2006 to November 2007, 25 patients with locally advanced non metastatic pancreatic cancer were treated by preoperative gemcitabine based chemo-radiotherapy. The radiation dose was 54 Gray in 30 fractions over 6 weeks prescribed to the isocenter. Gemcitabine (300 mg/m2) was given through a 30 minute intravenous infusion. This was done 30 minutes before the radiation sitting on a weekly basis throughout the radiotherapy course.

Approximately 6 weeks after the completion of chemo radiation, an evaluation was performed regarding tumour response and resectability as well as acute toxicity. Pancreaticoduodenectomy was performed for operable patients with surgical reconstruction.

**Results:**

Patients who achieved complete resection (CR) numbered 2 (8%), while those achieving partial resection (PR) totalled 11 (44%); six of these patients were considered ro be operable. Thus Pancreaticoduodenectomy was performed on 8 patients (2 with CR and 6 with PR) with surgical reconstruction. Patients who had a stable disease numbered 4 (16%), and those with progressive diseases included a group of eight (32%). The postoperative 30 day mortality occurred only in one patient (12.5%). Acute toxicity of chemoradiation occurred in the form of grade I leucopoenia and thrombocytopenia. Hepatic toxicity, nausea, and vomiting were found in 8 patients (32%), 10 patients (40%) and 4 patients (16%), respectively. The postoperative 30 day mortality occurred only in 1 patient. Also, minor biliary leakage and leakage from gastrointestinal anaestomosis both occurred in a single patient. Out of the 8 patients who underwent radical surgical resection, only one developed local recurrence and simultaneous liver metastasis during the follow up period. The median survival of all patients was 12 months.

**Conclusion:**

Preoperative gemcitabine based chemoradiation might benefit patients with locally advanced non metastatic pancreatic cancer by increasing the resectability without significant acute toxicity.

## Introduction

Almost 30% of patients with pancreatic cancer have locally advanced tumours in absence of distant metastasis. Because surgical resection is often contraindicated by vascular invasion, this condition has a dismal prognosis [1]. Therefore the role of preoperative chemoradiation in treatment of locally advanced non metastatic pancreatic cancer was investigated to help increase the rate of tumour resectability. Up to now, 5-flurouracil has been considered the standard agent for concurrent chemradiotherapy [2]. The combination of gemcitabine with concurrent radiation therapy is a promising new approach that is being investigated for treating patients' unresectable pancreatic cancer [3]. The aim of this study was to asses whether preoperative gemcitabine based chemo-radiotherapy increases the resectability rate for patients with locally advanced pancreatic adenocarcinoma without distant metastasis.

## Patients and methods

The current prospective study included 25 patients with locally advanced non metastatic pancreatic cancer and was carried out at radiation oncology and surgical oncology departments, South Egypt Cancer Institute, Assiut University during the period from March 2006 to November 2007. Informed consent was given by every patient who participated in this study, and the study was approved by our ethical committee.

### Inclusion criteria

The study included patients having pancreatic cancer with the following eligibility criteria: patients less than 70 years of age; patients with an ECOG performance status index which is at most 2; patients with surgically unresectable pancreatic carcinoma, or T4 disease (i.e. tumour encasement of celiac or superior mesenteric arteries as seen by CT scans and Doppler studies).

### Work up

Every patient in the study was subject to a history checks, physical examinations, laboratory investigations including tumour markers (CEA and CA19-9), and radiological studies (chest x-rays, abdominopelvic CT scans with contrast enhanced triple phase helicals, and Doppler studies). For jaundiced patients, ERCP and stent were performed.

All patients were treated by preoperative gemcitabine based chemo-radiotherapy.

• Radiotherapy (conformal radiotherapy): Each patient lied in a supine position on the flat table of the CT scan machine with radio-opaque markers in the midline and both sides of the patient defining the reference isocenter. Multiple CT slices of 0.5 cm intervals were taken through out the treatment volume. CT data were then transferred to the computer planning system. On each slice, a planning target volume was defined and included the gross disease as seen by CT scan. Two 0.5 cm safety margins were put in place to account for microscopic extension and for set up uncertainties. A 3D plan was then created taking into consideration the ICRU 50 recommendations and dosimetric limits for hepatic, renal and spinal cord toxicity criteria (i.e. the mean doses to the whole liver and to each kidney did not exceed 30 Gray, and 18 Gray respectively). The maximum dose to any point the in spinal cord did not exceed 45 Gray. All patients were treated by a photon beam of 15 MV in energy generated from a linear accelerator. The dose was 54 Gray in 30 fractions over 6 weeks (180 centi-Grays per fraction) prescribed to the isocenter.

• Chemotherapy: Each patient was given gemcitabine (300 mg/m2) through a 30 minute intravenous infusion. This occurred 30 minutes before the radiation sitting on a weekly basis throughout radiotherapy course.

### Evaluation

Approximately 6 weeks after the completion of chemo radiation, an evaluation was performed by physical examinations, tumour markers, and radiological studies, including abdominopelvic CT scans and Doppler studies. Each patient was evaluated regarding tumour response and resectability as well as acute toxicity according to the WHO common toxicity criteria, 1998.

### Surgery

At the time of surgery, abdominal laparoscopy was done for the 2 patients with CR and the patients with PR and no encasement of SM vessels to find peritoneal and or mesenteric metastases undetected by imaging. Thus, these patients were excluded from surgical exploration. At time of exploration, bilateral subcostal incisions were done for operable patients who underwent a pancreaticoduodenectomy (Whipple resection). Surgical reconstruction was done by pancreatic anastomosis; six patients underwent a pancreaticogastrostomy, where antrectomy of the stomach was done to leave a large residual stomach for insertion of the pancreatic stump into the gastric lumen. The pancreatic remnant was freed from the retroperitoneal space to provide about 3 cm of opening. A corresponding transverse opening was made on the posterior gastric wall. Only two patients underwent classical pancreaticojejunostomy.

## Results

### Patients' characteristics

The age of our patients ranged between 40 and 68 years with a median of 46 years. Males constituted 60% of patients with a male to female ratio of 1.5 to 1. According to the ECOG performance status, 5 (20%) patients had a score of 0, 15 (60%) had score of 1 and 5 (20%) had score of 2. The main presenting symptom was abdominal pain (20 out of 25 patients; 80%) while jaundice, GIT symptoms, fatigue and weight loss were reported in 60%, 40%, 32% and 28% of patients, respectively. Regarding TNM staging, most of our patients (22 out of 25 patients, 88%) showed a tumour size of ≥ 2 cm and only 10 patients (40%) showed radiological evidence of regional LN enlargement, table 1.

**Table 1:** Patients' characteristics

***Variable***	***No***	***%***
1- Age at diagnosis:		
• 40–49 years	14	56
• 50–69 years	11	44

2- Sex:		
• Male	15	60
• Female	10	40

3- Performance status (ECOG system):		
• Score 0	5	20
• Score 1	15	60
• Score 2	5	20

4- Presenting symptoms:		
• pain	20	80
• Jaundice	12	60
• GIT symptoms	10	40
• Fatigue	8	32
• Weight loss	7	28

5- Tumour size:		
• <2 cm	3	12
• ≥ 2 cm	22	88

6- LN status:		
• LN negative	15	60
• LN positive	10	40

**Total**	**25**	**100**

### Response after chemoradiation

Evaluation of response after chemoradiotherapy showed that only 2 out of 25 patients achieved CR (8%) while those achieving PR numbered 11 out of 25 (44%). Three of these patients still showed encasement of SMVs, and 2 patients out of the remaining 8 patients with no SMV encasement showed laparoscopic evidence of peritoneal and/or mesenteric seedling and thus were excluded from exploration. The other 6 patients with PR as well as the 2 patients with CR were evaluated and underwent radical surgery. Patients who had a stable disease totalled 4 out of 25 (16%), and those with progressive diseases numbered 8 out of 25 (32%), table 2.

**Table 2:** Response to chemoradiation and tumour respectability

***Variable***	***No***	***%***
CR	2 (Resectable)	8

PR	11 (6 patients of them were resectable)	44

SD	4	16

PD	8	32

**Total**	**25**	**100**

### Tumour respectability

**Figure 1:**
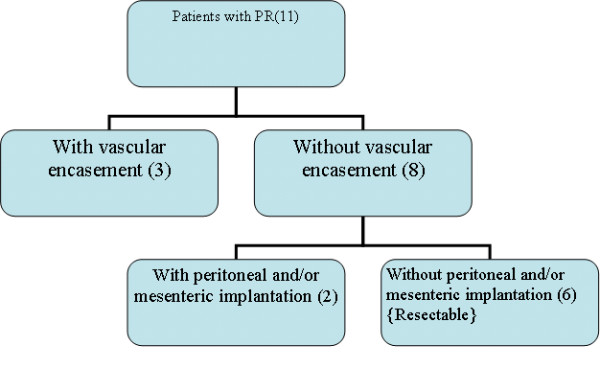
**Treatment profile of patients with partial response**.

Only 8 patients (32%) were considered respectable (underwent Whipple's operation) and included those who achieved CR in response to preoperative chemoradiation (2 patients) and those who achieved PR with no radiological evidence of SMV encasement and with no laparoscopic evidence of peritoneal and/or mesenteric seedling (6 patients), table 2 and figure 1. Prognostic factors which might predict tumor resectability were studied, table 3. Tumour size was the only factor which significantly affected pancreatic cancer resectability (P = 0.024).

**Table 3:** Factors which might predict tumor resectability:

	***Resected cases***		***Unresected cases***		
***Variable***	**No**	**%**	**No**	**%**	***P value***

1-Age at diagnosis:					
• 40–49 years	5	20	9	36	P > 0.05*
• 50–59 years	3	12	8	32	

2-Sex:					
• Male	5	20	10	40	P > 0.05*
• Female	3	12	7	28	

3-Performance status:					
• Score 0	3	12	2	8	
• Score 1	4	16	11	44	P > 0.05*
• Score 2	1	4	4	16	

4-Tumour size:					
• <2 cm	3	12	0	0	P = 0.024**
• ≥ 2 cm	5	20	17	68	

5-LN status:					
• LN negative	5	20	10	40	P > 0.05*
• LN positive	3	12	7	28	

• *Insignificant difference p > 0.05

• ** Significant difference p < 0.05

### Treatment related toxicities and postoperative morbidity and mortality

Toxicities of preoperative chemoradiation were mainly haematological, where grade 1 leucopoenia and thrombocytopenia were found in 8 out of 25 patients (32%), grade 1 hepatic toxicity was discovered in 10 patients (40%), and grade 1 nausea and vomiting occurred in 4 patients (16%). The postoperative 30 day mortality occurred only in one patient (12.5%) due to a reactionary haemorrhage from the portal vein. The postoperative morbidity occurred in the form of minor biliary leakage (1 patient, 12.5%) and leakage from gastrointestinal anastomosis (1 patient, 12.5%) that healed conservatively. Delayed gastric emptying occurred in 3 patients (37.5%).

### Disease relapse and survival, figure 2

**Figure 2:**
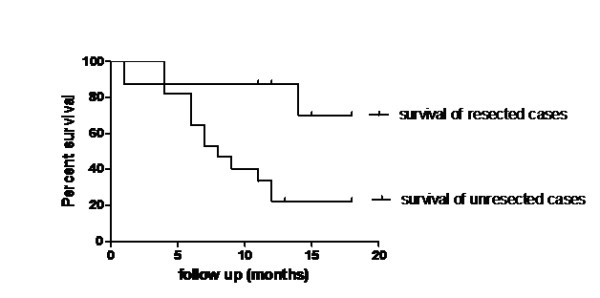
**Overall survival curves in both resected and unresected cases**.

Out of 8 patients who underwent radical surgical resection, one patient died one month after surgery, and one patient (12.5%) developed local recurrence and simultaneous liver metastasis during the follow up period and eventually died after a follow up of 14 months. The median survivals were 12 months and 8 months for all patients and unresected patients, respectively. The survival curves for resected and unresected patients are significantly different and favour resected cases, with the 1 year survival rates being 87.5% and 22.4% for resected and unresected cases, respectively (P = 0.02).

## Discussion

Improved resectability is a major theoretical benefit of preoperative chemoradiation for pancreatic cancer [4]. The combination of gemcitabine with concurrent radiation therapy is a promising new approach that is being used in patients with unresectable pancreatic cancer [3].

The age and sex distribution in the present study showed that the median age of patients was 46 years with male to female ratio of 1.5:1. These data are matched with Egyptian figures [5] and also matched with western ones [6].

Pancreatic tumours with the greatest dimension (≥ 2 cm) were present in most of cases (22 patients; 88%). This figure is higher than the one reported by **Maheshwari and Moser **[1] who found that only 30% of patients with pancreatic cancer have large, locally advanced tumors and non metastatic disease. This difference may be partly due to a lack of routine check up procedures in developing countries (including Egypt) and partly due to the delay of attendance of most cases to the cancer institute.

About two thirds of patients in this study (17 out of 25; 68%) showed radiographic evidence of response to preoperative chemoradiation: Two of them showed CR, 11 showed PR, and 4 had a stable disease. These figures are comparable with those found by **White et al**. [4] where 64% of patients had decreased or stable primary tumour sizes after neoadjuvant therapy. The remission rate (CR & PR) in the present study was 52% (13 out of 25 patients) which was lower than that reported by **Wilkowshi et al**. [2] who observed a 69% remission rate. This higher remission rate may be due to the addition of weekly cisplatin in the chemotherapy given in the reported study. On the other hand, our figures are higher than those reported by **Smeenk et al**. [7] who stated that only 42% (16 out of 38) of patients showed radiographic evidence of response (26% PR & 16% SD). This difference may result from the use of fluorouracil and not gemcitabine based chemoradiation in the reported series in constrast to our study, where gemcitabine based chemoradiotherapy was used.

The present study showed that 8 out of 25 patients (32%) with locally advanced pancreatic cancer were surgically resectable (2 patients of CR and 6 of PR with no radiographic evidence of SMV encasement and no laparoscopic evidence of peritoneal or mesenteric implantation). This figure is comparable to that reported by **Hoffman et al**. [8] and **Wilkowshi et al**. [2] who found that the resectability rates were 33% and 30%, respectively. However, our figure is slightly lower than that reported by **Brunner et al**. [9] where 37% (10 out of 37) of patients were considered resectable. This slight difference may be due to the use of different chemotherapy regimen (mitomycin C in addition to fluorouracil) concurrently with radiotherapy in the reported study. Among factors which might predict tumor resectability, tumor size was the only significant one. This is in agreement with **Reber **[10] who stated that small pancreatic tumors (<2 cm diameter) are more likely be resectable than larger ones.

Regarding the toxicity to preoperative chemoradiation, the incidences of grade I haematological toxicity (32%) and grade 1 nausea and vomiting (16%) in our series were lower than those reported by **Bruner et al**. [9] where grade 3 haematological toxicity was 30% and grade 3 nausea and vomiting was 20%. The higher reported toxicity may have occurred from the use of different chemotherapy regimen which consisted of 5-flurouracil and mitomycin concurrently with radiotherapy. In contrast to our study, most of the reported trials using gemcitabine based chemoradiotherapy showed higher toxicity profiles, where grade 3 hematological and nonhematological toxicities were detected in 36% and 5% of patients, respectively [11]. This could be explained by the use of gemcitabine doses of 1000 mg/m2 given on days 1, 8 and 15 every 4 weeks in the reported series.

The incidence of disease relapse in this study (12.5%) was similar to that found by the **Lee Moffitt cancer centre **(12.5%) [12]. Two randomised trials evaluated chemoradiation alone with radiation therapy and found that the mean survival time was significantly increased from 6.3 months to 10.4 months when 5-FU was added to radiotherapy (p < 0.05). In addition, the median survival time was increased from 5.6 months to 8 months [13,14]. The median survival among our patients was 12 months, which was higher than that reported with radiotherapy with concurrent 5-FU. This is consistent with the results of a randomised trial conducted by **Li et al**. [15], who compared radiotherapy with concurrent gemcitabine (18 patients) based on the same radiation therapy regimen with 5-FU (16 patients). They found a statistically significant median survival advantage in favour of the gemcitabine group (14.5 versus 6.7 months; p < 0.027).

Our results are confirmed by a meta-analysis examining the data from 29 randomised trials (including 3458 patients), and they support the notion that 5-FU based chemotherapy regimens show better survival outcomes over the best supportive care (<0.0001), and that gemcitabine based chemotherapy regimens show better survival outcomes over 5-FU based regimens (<0.005) [16], table 4.

**Table 4:** Results of Fung et al., 2003, meta-analysis.

*No of trials*	*regimens*	*No of patients*	*Average median an survival(months)*	*Hazard ratio*	*p-value*
9	Best supportive care vs. 5-FU based combinations	434 262	3.87 6.38	0.53	<0.0001

7	5-FU/other agent alone vs. 5-FU based combination	428 414	5.23 4.98	0.88	0.1

3	5-FU based combination vs. 5-FU based combination	121 121	3.75 4.38	0.85	0.25

1	5-FU vs. Gemcitabine	63 63	4.41 5.65	0.56	<0.005

2	Miscellaneous new agent vs. Gemcitabine	241 242	3.70 6.08	0.61	<0.0001

***7***	Gemcitabine vs. Gemcitabine based combination	758 745	6.62 6.98	0.92	0.15

The median survival in the current study is comparable to that of the most reported series [10,17,18] which reported median survivals of 11 months, 13 months, and 15.4 months, respectively. In the present study, the resected cases showed significantly higher survival rates than that of the unresected cases, where the 1 year survival rates were 87.5% and 22.4% for resected and unresected cases, respectively (P value; 0.02 and Hazard Ratio; 0.26 with 95% CI; 0.08 to 0.82). These figures are comparable to the reported series where the 1 year survival rates were 87.5% in the resected cases [19].

## Conclusion

Although overall survival remains poor, treatment with preoperative gemcitabine based chemoradiation might benefit patients with locally advanced non metastatic pancreatic cancer by helping to increase resectability without significant acute toxicity.

## Competing interests

The authors declare that they have no competing interests.

## Authors' contributions

MA and ME carried out the patient diagnosis, investigation, management, and follow up. DM and AAA-E carried out the patient diagnosis, investigation, management, follow up, general coordination, drafting of the manuscript, and writing of the final manuscript. All authors have read and approved the final manuscript.

## References

[B1] Maheshwari V, Moser J (2005). Current management of locally advanced pancreatic cancer. Nature Clinical Practice Gastroenterology & Hepatology.

[B2] Wilkowski R, Thoma M, Weingandt H, Dühmke E, Heinemann V (2005). Chemoradiation for ductal pancreatic carcinoma: Principles of combining chemotherapy with radiation, definition of target volume and radiation dose. JOP.

[B3] Ammori JB, Colletti LM, Zalupski MM, Eckhauser FE, Greenson JK, Dimick J, Lawrence TS, McGinn CJ (2003). Surgical resection following radiation therapy with concurrent gemcitabine in patients with previously unresectable adenocarcinoma of the pancreas. Journal of Gastrointestinal Surgery.

[B4] White R, Lee C, Anscher M, Gottfried M, Wolff R, Keogan M, Pappas T, Hurwitz H, Tyler D (1999). Preoperative chemoradiation for patients with locally advanced adenocarcinoma of the pancreas. Annals of Surgical Oncology.

[B5] El-Hattab OH, Nouh MA (1998). Epidemiology of cancer. NCI.

[B6] Gold EL, Goldi SB (1998). Epidemiology of and risk factors for pancreatic cancer. Surg Oncol Clin N Am.

[B7] Smeenk HG, de Castro SM, Jeekel JJ, Kazemier G, Busch OR, Incrocci L, Erdmann J, Hop WC, Gouma DJ, van Eijck CH (2005). Locally advanced pancreatic cancer treated with radiation and 5-fluorouracil: A first step to neoadjuvant treatment?. Digestive Surgery.

[B8] Hoffman JP, Weese JL, Solin LJ, Engstrom P, Agarwal P, Barber LW, Guttmann MC, Litwin S, Salazar H, Eisenberg BL (1995). A pilot study of preoperative chemoradiation for patients with localized adenocarcinoma of the pancreas. Am J Surg.

[B9] Brunner TB, Grabenbauer GG, Kastl S, Herrmann O, Baum U, Fietkau R, Klein P, Bautz W, Schneider T, Hohenberger W, Sauer R (2000). Preoperative chemoradiation in locally advanced pancreatic carcinoma: A phase II study. Onkologie.

[B10] Reber HA (1995). Small pancreatic tumors: Is size an indication of curability?. Journal of Hepato-Biliary-Pancreatic Surgery.

[B11] Yamazaki H, Nishiyama K, Koizumi M, Tanaka E, Ioka T, Uehara H, Iishi H, Nakaizumi A, Ohigashi H, Ishikawa O (2007). Concurrent chemoradiotherapy for advanced pancreatic cancer: 1000 mg/m2 gemcitabine can be administered using limited field radiotherapy. Strahlenther Oncol.

[B12] Lee Moffitt Cancer Center and Research Institute (2000). Treatment of resectable and locally advanced pancreatic cancer. Cancer control.

[B13] Mortel CG, Childs DS, Reitemeier RJ, Colby MY, Holbrook MA (1969). Combined 5-flurouracil and supervoltage radiation therapy of locally unresectable gastrointestinal cancer. Lancet.

[B14] Gastrointestinal Tumour Study Group (1981). Therapy of localised unresectable pancreatic carcinoma: a randomised comparison of high dose ((6000 rads)) radiation alone, moderate dose (4000 rads) radiation + 5-FU and high dose radiation + 5-FU. Cancer.

[B15] Li CP, Chao Y, Chi KH, Chan WK, Teng HC, Lee RC (2003). Concurrent chemoradiotherapy treatment (CCRT) of locally advanced pancreatic cancer (LAPC): gemcitabine (GEM) versus 5-flurouracil (5-FU) [abstract]. Proc Ann Meet Am Soc Clin Oncol.

[B16] Fung MC, Ishiguro H, Takayama S, Morizane T, Adachi S, Skata T (2003). Survival benefit of chemotherapy treatment in advanced pancreatic cancer: a meta-analysis [abstract]. Proc Ann Meet Am Soc Clin Oncol.

[B17] Kim HJ, Czischke K, Brennan MF, Conlon KC (2002). Does neoadjuvant chemoradiation downstage locally advanced pancreatic cancer?. J Gastrointest Surg.

[B18] Massucco P, Capussotti L, Magnino A, Sperti E, Gatti M, Muratore A, Sgotto E, Gabriele P, Aglietta M (2006). Pancreatic resections after chemoradiotherapy for locally advanced ductal adenocarcinoma: Analysis of perioperative outcome and survival. Annals of Surgical Oncology.

[B19] Takai S, Satoi S, Yanagimoto H, Toyokawa H, Takahashi K, Terakawa N, Araki H, Matsui Y, Sohgawa M, Kamiyama Y (2008). Neoadjuvant chemoradiation in patients with potentially resectable pancreatic cancer. Pancreas.

